# FABP4 Induces Vascular Smooth Muscle Cell Proliferation and Migration through a MAPK-Dependent Pathway

**DOI:** 10.1371/journal.pone.0081914

**Published:** 2013-11-29

**Authors:** Josefa Girona, Roser Rosales, Núria Plana, Paula Saavedra, Lluís Masana, Joan-Carles Vallvé

**Affiliations:** Research Unit on Lipids and Atherosclerosis, “Sant Joan” University Hospital, Universitat Rovira i Virgili, IISPV, Spanish Biomedical Research Centre in Diabetes and Associated Metabolic Disorders, Reus, Spain; Northwestern University, United States of America

## Abstract

**Purpose:**

The migration and proliferation of vascular smooth muscle cells play crucial roles in the development of atherosclerotic lesions. This study examined the effects of fatty acid binding protein 4 (FABP4), an adipokine that is associated with cardiovascular risk, endothelial dysfunction and proinflammatory effects, on the migration and proliferation of human coronary artery smooth muscle cells (HCASMCs).

**Methods and Results:**

A DNA 5-bromo-2′-deoxy-uridine (BrdU) incorporation assay indicated that FABP4 significantly induced the dose-dependent proliferation of HCASMCs with a maximum stimulatory effect at 120 ng/ml (13% vs. unstimulated cells, p<0.05). An anti-FABP4 antibody (40 ng/ml) significantly inhibited the induced cell proliferation, demonstrating the specificity of the FABP4 proliferative effect. FABP4 significantly induced HCASMC migration in a dose-dependent manner with an initial effect at 60 ng/ml (12% vs. unstimulated cells, p<0.05). Time-course studies demonstrated that FABP4 significantly increased cell migration compared with unstimulated cells from 4 h (23%vs. 17%, p<0.05) to 12 h (74%vs. 59%, p<0.05). Pretreatment with LY-294002 (5 µM) and PD98059 (10 µM) blocked the FABP4-induced proliferation and migration of HCASMCs, suggesting the activation of a kinase pathway. On a molecular level, we observed an up-regulation of the MAPK pathway without activation of Akt. We found that FABP4 induced the active forms of the nuclear transcription factors c-jun and c-myc, which are regulated by MAPK cascades, and increased the expression of the downstream genes cyclin D1 and MMP2, CCL2, and fibulin 4 and 5, which are involved in cell cycle regulation and cell migration.

**Conclusions:**

These findings indicate a direct effect of FABP4 on the migration and proliferation of HCASMCs, suggesting a role for this adipokine in vascular remodelling. Taken together, these results demonstrate that the FABP4-induced DNA synthesis and cell migration are mediated primarily through a MAPK-dependent pathway that activates the transcription factors c-jun and c-myc in HCASMCs.

## Introduction

The proliferation and directed migration of abnormal vascular smooth muscle cells (VSMCs) from the media into the intima play major roles in the pathogenesis of atherosclerotic lesions, the occurrence of restenosis after angioplasty, and the accelerated arteriopathy after cardiac transplantation[1]. Furthermore, the activation of VSMCs is a key event in the formation of the fibrous cap and the neointima. These processes are triggered by multiple cytokines and growth factors, such as tumour necrosis factor-α (TNF-α), platelet-derived growth factor (PDGF), insulin-like growth factor-I (IGF-I), and transforming growth factor-β (TGF-β), among others, and mitogen-activated protein kinase (MAPK) and phosphatidylinositol 3-kinase (PI3K)/Akt are the two major signalling pathways linked to migration and proliferation[2,3]. Understanding the potential mechanisms governing VSMC migration and proliferation may provide new perspectives in the effort to inhibit this inflammatory process.

The adipose fatty acid-binding protein (FABP), also known as FABP4 and aP2, is one of the most well-characterised intracellular lipid transport proteins[4]. It belongs to a superfamily of low-molecular-weight intracellular lipid-binding proteins and plays a central regulatory role in energy metabolism and inflammation[5-7]. FABP4 is highly expressed in mature adipocytes and accounts for approximately 6 % of the soluble protein in the adipocyte. FABP4 is also found in circulating blood plasma. In the last several years, much effort has been focused on uncovering the role of FABP4. However, neither the secretory pathways nor the functions of circulating FABP4 are known. We and other authors have shown that FABP4 levels are increased in obesity, metabolic syndrome (MS), type 2 diabetes (T2D), and familial combined hyperlipidaemia or lipodystrophy syndromes and that these increased levels are also closely correlated with adverse lipid profiles and insulin resistance[8-14]. In these and other studies, serum FABP4 predicted the development of MS and atherosclerosis[15-17]. Moreover, increased plasma levels of FABP4 in non-elderly men were independently associated with the presence of coronary artery disease[18]. In addition, FABP4 is found in human atherosclerotic plaques, and its presence is associated with high-risk atherosclerotic plaques such as unstable, inflammatory and vulnerable plaques[19-22]. 

FABP4 has been implicated in several critical cellular processes, such as the uptake and intracellular storage of fatty acids and the regulation of gene expression, cell proliferation, and differentiation[23]. In addition to being expressed in adipocytes and macrophages, the constitutive or induced expression of FABP4 has been found in coronary endothelial cells, trophoblasts, muscle cells and epithelial cells, suggesting additional biological roles[24,25]. A recent study demonstrated that FABP4 decreased the contractility of myocardial muscle cells, which suggests that the release of FABP4 into the bloodstream could have a direct effect on some peripheral cells and tissues[26]. In addition, we recently demonstrated that high levels of plasma FABP4, as well as other inflammation mediators, were associated with endothelial dysfunction as assessed by peripheral artery tonometry[27,28], and in an in vitro study, we previously demonstrated that recombinant FABP4 causes endothelial dysfunction by impairing the insulin-signalling pathway and NO production[29]. Furthermore, the elevated expression of intracellular FABP4 in endothelial cells was found to contribute to the dysfunction of these cells by reducing eNOS[30]. The knockdown of FABP4 in endothelial cells significantly reduces the proliferation of these cells both under baseline conditions and in response to VEGF and bFGF[24]. Although all of these data suggest a role for FABP4 in vascular dysfunction, there are no data regarding the effect of FABP4 on human coronary artery smooth muscle cell (HCASMC) activation.

These data, along with our own observations illustrating the influence of circulating FABP4 on vascular function, support testing the hypothesis that the high levels of circulating FABP4 in altered metabolic conditions could modify the normal function of VSMCs and cause proliferation and migration. The aims of the current study were to evaluate the direct influence of FABP4 on HCASMC proliferation and migration and to analyse the intracellular signalling pathways involved. 

## Material and Methods

### Cell culture and reagents

Primary HCASMCs were obtained from Cascade BiologicsTM (Invitrogen Life Technologies, Paisley, UK). After thawing, the cells were seeded into 75 cm^2^ flasks and cultured according to the supplier’s recommendations in Medium 231. Medium 231 was supplemented with smooth muscle growth supplement (SMGS) and 1% gentamicin/amphotericin solution (Invitrogen Life Technologies, Paisley, UK). The supplemented medium contained foetal bovine serum (4.9 % v⁄v final concentration), human basic fibroblast growth factor (2 ng⁄ml), human epidermal growth factor (0.5 ng⁄ml), heparin (5 ng⁄ml), insulin (5 µg⁄ml) and BSA (0.2 µg⁄ml). The cells were placed in a humidified incubator at 37°C and 5% CO_2_ until there were enough cells available for the experiments. In the current study, the HCASMCs were used at passage 6. Before the initiation of the assays, HCASMCs that were in exponential growth were switched into Medium 231 supplemented with 0.1% FBS in the absence of growth factors for 24 h to achieve cell quiescence.

 The cytotoxicity assay was performed by analysing LDH release into the medium using the Cytotoxicity Detection Kit (Roche Diagnostics, Basel, Switzerland). TNFα was purchased from Calbiochem (Merck, Darmstadt, Germany). To study FABP4 activation pathways in HCASMCs, the PI3K inhibitor LY294002 and the MAPK inhibitor PD98059 (Calbiochem-Merck, Darmstadt, Germany) and the c-Jun inhibitor SP600125 and the c-Myc inhibitor 10074-G5 (Sigma-Aldrich, Madrid, Spain) were used. Human recombinant FABP4 and the anti-FABP4 antibody were obtained from BioVendor (Brno, Czech Republic). The anti-Akt, anti-pAkt (Ser^473^), anti-p44/p42 MAPK (Erk1/2), anti-phospho-p44/p42 MAPK (Erk1/2) Thr^202^/Tyr^204^, anti-NF-kB p65 and anti-cyclin D1 antibodies were obtained from Cell Signaling Technology, Inc. (Beverly, MA, USA). Antibodies specific for the active forms of the following transcription factors were used: phospho-ATF2 (Thr^71^), phospho-c-jun (Ser^73^), c-myc and STAT1α were purchased from Active Motif (La Hulpe, Belgium). Anti-actin was purchased from Santa Cruz Biotechnology (Santa Cruz, CA, USA), and IgG-HRP was purchased from Dako (Glostrup, Denmark).

### Cell proliferation studies

Cell proliferation was analysed by measuring DNA synthesis with a colorimetric bromodeoxyuridine (BrdU) enzyme-linked immunosorbent assay (ELISA) kit (Roche Diagnostics, Basel, Switzerland), according to the manufacturer’s instructions. Briefly, 1x10^4^ cells were seeded into a 96-well microplate and cultured with or without FABP4 (30-120 ng/ml), TNF-α (10 ng/ml), anti-FABP4 antibody (40 ng/ml) and/or LY294002 (5 µM), PD98059 (10 µM), 10074-G5 (10 µM) and SP600125 (10 µM) for 24 h. The cells were then labelled with BrdU labelling reagent for 10 h. After fixation, the cells were incubated with anti-BrdU antibody for 90 min. After washing, 100 μl of substrate (tetramethylbenzidine) was added to each well, and the plates were incubated at room temperature for 30 min. The absorbance at 450 nm was measured with an ELISA reader (Synergy H4, Biotek, USA).

### *In vitro* wound-healing assay

Cell migration was analysed with the in vitro scratch assay[31]. The cells were cultured in 12-well plates, and after the induction of quiescence, a single scratch wound was created in the centre of the cell monolayer by the gentle removal of the attached cells with a sterile plastic pipette tip. The cells were incubated with FABP4 (30-240 ng/ml) and/or LY294002 (5 µM), PD98059 (10 µM), 10074-G5 (10 µM) and SP600125 (10 µM) for 24 h in serum-reduced Medium 231 (containing 0,1 % foetal calf serum in the absence of growth factors). Images of the cells migrating into the wound were taken at 0 h and then every 2 h until the scratch wound was closed at 24 h; the images were compared to quantify the migration rate of the cells. The closure of the wound was considered to represent 100% migration. The cell images were captured using a microscope (Olympus IX71, Spain) and analysed using imaging software (Xcell). To investigate the effect of FABP4 on fibulin 4, fibulin 5, MMP2, and CCL2 expression, migrating HCASMC were incubated with 120 ng/ml of FABP4 in the presence or absence of c-myc (10074G5) and c-jun (SP600125) inhibitors for 24 h. Briefly, the cells were plated in 8.8-cm^2^ culture dishes. After the induction of quiescence, a cross scratch wound was created by the gentle removal of the attached cells with a sterile plastic Pasteur pipette. The cells were incubated with FABP4 with or without inhibitors, and 24 h later, the migrating cells were scraped from the plate under microscopic observation.

### Total cellular and nuclear extracts

To obtain total cellular extracts, HCASMCs were cultured in 10 cm culture dishes until the cells reached 90% confluence. After the induction of quiescence, the cells were incubated with FABP4 (120 ng/ml) or TNF (30 ng/ml) for 5, 15 or 30 min. At different time points, the cells were rinsed with ice-cold PBS and lysed in lysis buffer, which was composed of 50 mM Tris–HCl, 150 mM NaCl, 0.1% SDS, 1% Nonidet P-40, 0.5% deoxycholate and phosphatase inhibitors (Roche Diagnostics, Basel, Switzerland). To investigate the effect of FABP4 on cyclin D1 expression, proliferating HCASMC were incubated with 120 ng/ml FABP4 in the presence or absence of c-myc (10074G5) or c-jun (SP600125) inhibitors for 6 h. The cells were then lysed, and total extracts were analysed for cyclin D1 expression by western blotting. The cells were then stored at −80°C until they were processed. The total protein concentration was measured using a Bradford assay kit (BioRad, USA), and the immunoblot analysis was then performed. For the nuclear extracts, HCASMCs were cultured in 10 cm culture dishes until the cells reached 90% confluence. After the induction of quiescence, the cells were incubated with FABP4 (120 ng/ml) or TNF (30 ng/ml) for 15 min or 2 or 4 h. Nuclear protein extracts were prepared essentially as described below. The HCASMCs were homogenised in ice-cold hypotonic buffer (10 mM HEPES, 0.1 mM EDTA, 0.1 mM EGTA, 10 mM KCL, 0.75 mM spermidine, 0.15 mM spermine, 1 mM DTT and phosphatase inhibitors) and centrifuged at 16,000xg for 10 min at 4°C. The homogenate was layered onto extraction buffer (20 mM HEPES, 25% glycerol, 0.42 mM NaCl, 1 mM EDTA, 1 mM EGTA, 1 mM DTT, and phosphatase inhibitors) and centrifuged at 16,000×g for 60 min at 4°C. After centrifugation, the supernatant (nuclear extract) was collected, the protein concentration was measured with a Bradford assay kit (Bio-Rad, USA) and the immunoblot analysis was performed. The extracts were used immediately or stored at -80°C for later use.

### Immunoblot analysis

Electrophoresis and immunoblot analysis were performed using the NuPAGE protein analysis system (Invitrogen Life Technologies, UK). The membranes were blocked with a 2% ECL Advance Blocking Reagent (Amersham Biosciences, USA) and incubated with anti-FABP4, anti-cyclin D1, anti-actin, anti-Akt, anti-phospho-Akt (Ser^473^), anti-ERK1/2 and anti-phospho-p44/p42 MAPK (Erk1/2) Thr^202^/Tyr^204^ antibodies, as well as anti-phospho-ATF2 (Thr^71^), anti-phospho-c-jun (Ser^73^), anti-c-myc, anti-STAT1α and anti-NF-kB p65 antibodies. The antigen-antibody complexes were detected by incubating the membrane with an HRP-conjugated anti-IgG antibody. The bands were visualised using ECL reagents (Amersham Pharmacia, USA) with the VersaDoc image system and quantified with the Quantity One analysis software, version 4.6.2 (Bio Rad, USA). The relative levels of the phosphorylated forms of Akt and ERK1/2 were quantified after normalisation to the total protein levels, and the levels of the activated forms of the transcription factors were expressed relative to the levels of the respective factors in the non-stimulated cells at each time point; all of the values were expressed in arbitrary units (AU).

### Quantitative real-time RT-PCR

Total RNA was isolated from the cells using the ABI PRISM 6100 Nucleic Acid PrepStation (Applied Biosystems, CA, USA). The absorbance at 260 nm was used to measure the RNA concentration, and an absorbance ratio of 260/280 nm was used to analyse the quality of the RNA. Total RNA (0.5 μg) was reverse transcribed to cDNA using random hexamers and SuperScript II (Invitrogen Life Technologies, UK) following the manufacturer’s protocol. TaqMan primers and probes for fibulin 4, fibulin 5, MMP2 and CCL2 were obtained from validated and pre-designed Assays-on-Demand products (Applied Biosystems, CA, USA) and were used in real-time PCR amplifications. The mRNA expression for each gene and sample was calculated using the recommended 2-ΔΔCt method. The control group (untreated cells) was defined as the calibrator in this experiment. GAPDH was used as a housekeeping gene to normalise the results of the gene of interest.

### Statistical analysis

The results were represented as the means ± SEM of at least 3 separate experiments. Differences between the means were determined using a t test or a one-way analysis of variance (ANOVA), which was followed by a Dunnett’s post-hoc test for multiple comparisons. The differences were considered to be significant at p<0.05. The GraphPad Prism 5.0 software, GraphPad Software Inc. was used for the statistical analyses.

## Results

### Effect of FABP4 on human coronary artery smooth muscle cell (HCASMC) proliferation

**Figure 1:**
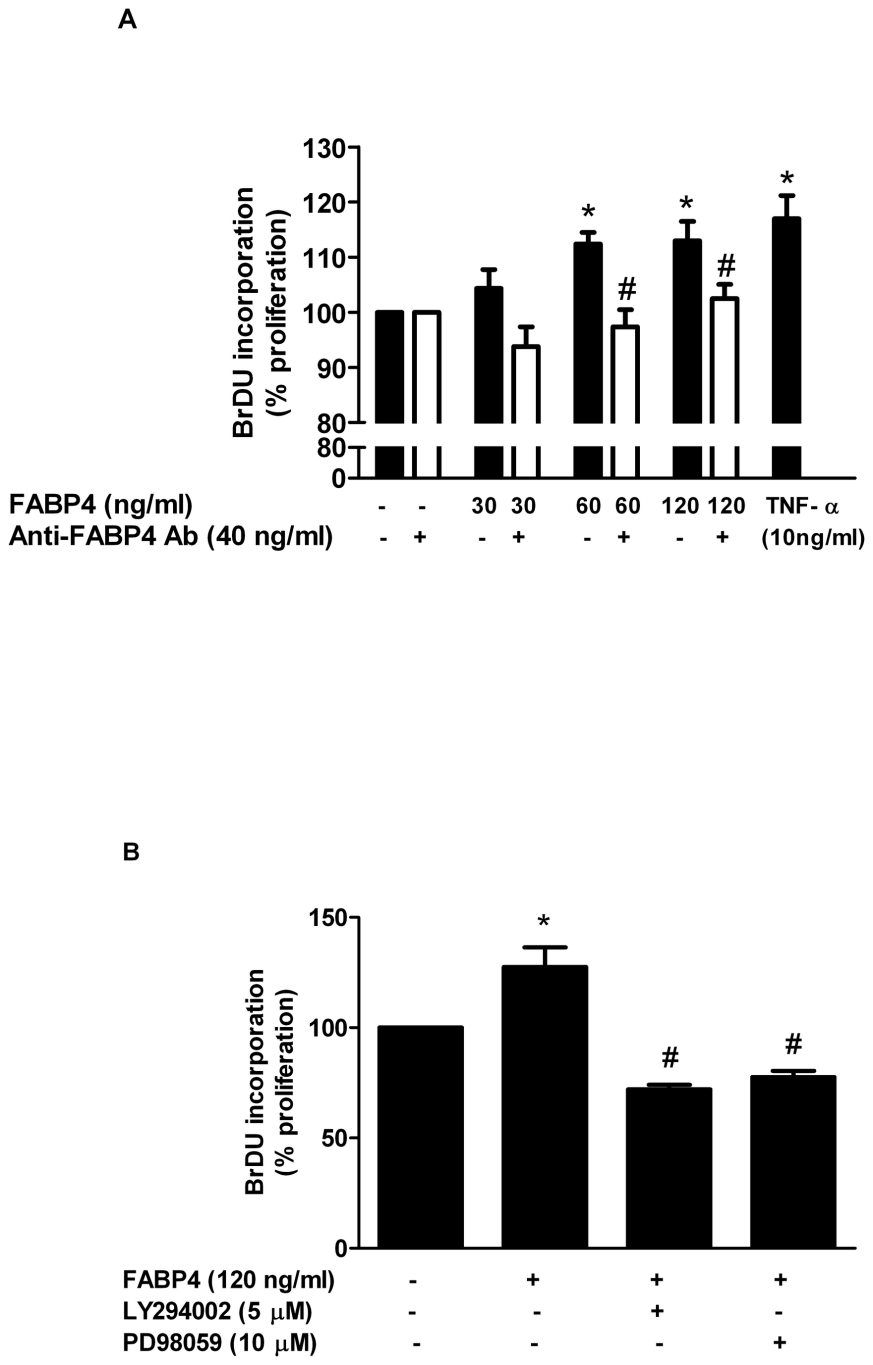
Effect of FABP4 on HCSMCs proliferation as assessed by BrdU incorporation. (A) Effect of FABP4 (30-120 ng/ml) with and without anti-FABP4 antibody (40 ng/ml) on the proliferation HCASMCs at 24 h. TNF-α (10 ng/ml) was used as a positive control. (B) Effect of LY294002 (5 µM) and PD98059 (10 µM) on FABP4 (120 ng/ml)-induced HCASMCs proliferation at 24 h. The results are expressed as the mean ± SEM of three experiments run in quadruplicate. *p<0.05 vs unstimulated cells; #p<0.05 vs FABP4-stimulated cells.

To assess the effect of FABP4 on HCASMC proliferation, we performed dose-response experiments using BrdU uptake as a marker for DNA synthesis. The BrdU incorporation indicated that, when HCASMCs were stimulated with increasing concentrations of FABP4 (30-120 ng/ml), a significant dose-response effect on cell proliferation at 24 h was observed (Figure 1). FABP4 had no effect on HCASMC proliferation at 30 ng/ml, while a significant increase in proliferation was observed at 60 ng/ml and 120 ng/ml (12% and 13% vs. unstimulated cells, respectively, p<0.05). TNF-α at 10 ng/ml, which was chosen as the positive control because of the well-known mitogenic effect of TNF-α on SMCs, resulted in an increase of 17% with respect to the unstimulated cells (p<0.05). Cell proliferation was also measured in the presence of an anti-FABP4 antibody (40 ng/ml) to analyse the specific effect of FABP4. The presence of the anti-FABP4 antibody completely inhibited the proliferation induced by FABP4 at concentrations of 60 ng/ml and 120 ng/ml (p<0.05) (Figure 1A). Because PI3K and MAPK pathways are the primary regulators of cell proliferation, we investigated the effects of LY294002, a selective inhibitor of PI3K and of PD98059, a selective inhibitor of MAPK, by BrdU uptake measurement. As shown in Figure 1B, LY294002 (5 µM) and PD98059 (10 µM) significantly blocked FABP4-induced HCASMC proliferation (p<0.05), suggesting that both pathways areinvolved in FABP4-induced cell proliferation. 

### Effect of FABP4 on human coronary artery smooth muscle cell (HCASMC) migration

**Figure 2:**
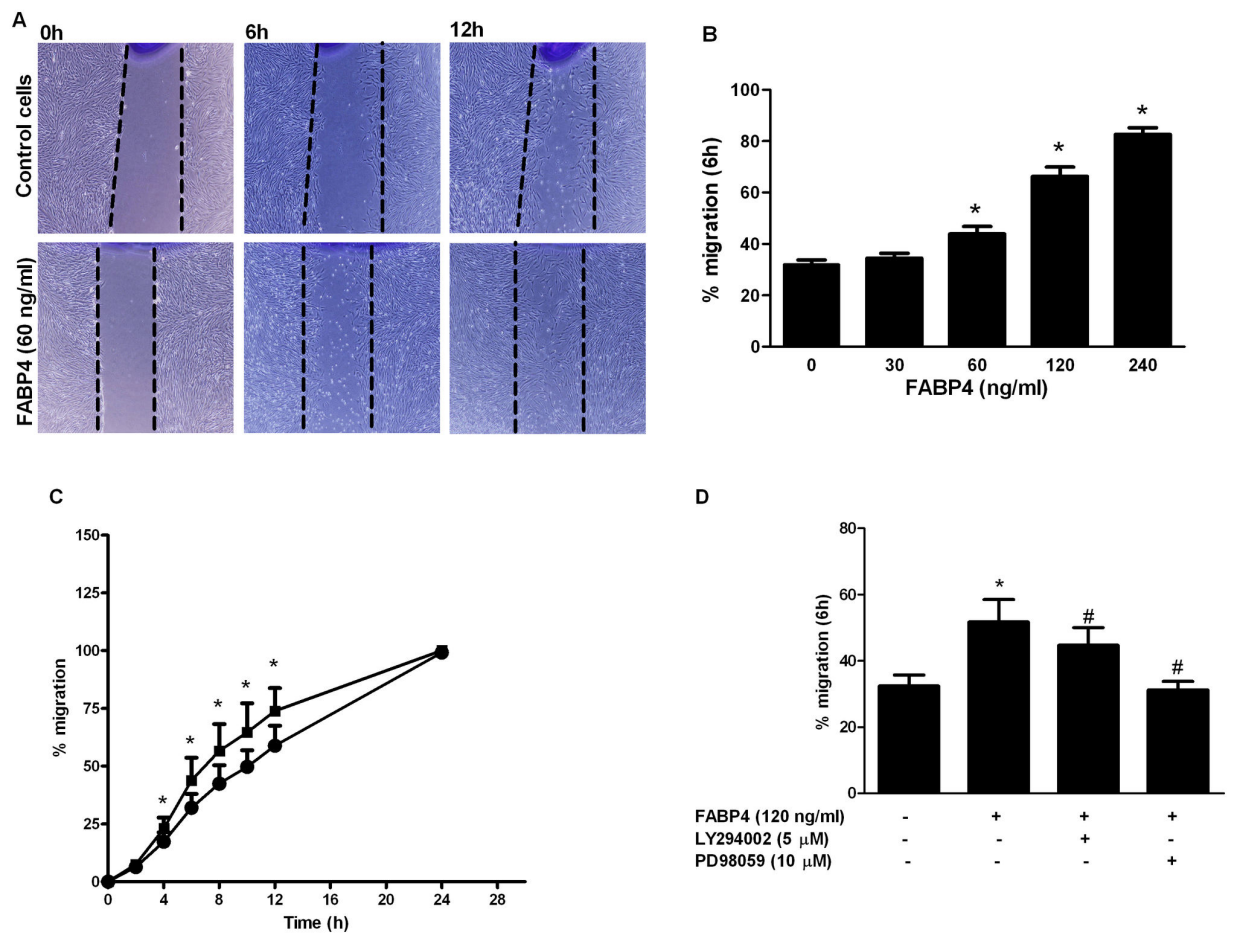
Effect of FABP4 on HCASMC migration. (A) An *in* *vitro* wound-healing assay indicates that FABP4 at 60 ng/ml induced HCASMC migration. Confluent cells were scratch wounded and allowed to migrate for 12 h. Representative images of the cells after 0 h, 6 h and 12 h of migration are shown. Photomicrographs of the cell images were obtained at 100x magnification in a phase-contrast microscope. (B) The effects of FABP4 (30-240 ng/ml) on HCASMC migration after 6 h of treatment. The results are shown as the percentage of increase in relation to the cells treated with medium alone (control). (C) A time-course (0-24 h) of the effect of 60 ng/ml FABP4 on HCASMC migration. (D) The effect of LY294002 (5 µM) and PD98059 (10 µM) on FABP4-induced HCASMCs at 6 h. *p<0.05 vs unstimulated cells; #p<0.05 vs FABP4-stimulated cells. The results are shown as a percentage of the migration rate. The results are expressed as the mean ± SEM of three experiments run in sextuplicate. *p<0.05 vs. unstimulated cells (control).

To address the effect of FAPB4 on HCASMC migration, a wound-healing assay was performed. We analysed the cell migration every 2 h for 24 h. As shown in Figure 2A, FABP4-treated (60 ng/ml) HCASMCs migrated earlier than the untreated cells and almost completely closed the denuded area after 12 h of treatment (Figure 2A). As shown in Figure 2B, dose-response studies (30-240 ng/ml) revealed that FABP4 significantly increased cell migration at 60 ng/ml, 120 ng/ml and 240 ng/ml with respect to the untreated cells at 6 h (12%, p<0.05; 34%, p<0.05; and 51%, p<0.05, respectively). As shown in Figure 2C, the time-course studies (0 h-24 h) revealed that FABP4 (60 ng/ml) significantly increased the migration of HCASMCs compared with the untreated cells, with a 6% increase within 4 h of treatment (p<0.05). This significant increase in cell migration was observed at each time point, with a maximum effect (an increase of 15%) after 12 h of treatment (p<0.05). Both treated and untreated cells reached 100% migration at the 24 h time point (Figure 2C). 

To investigate the roles of the PI3K and MAPK pathways in cell migration, HCASMCs were incubated with FABP4 (120 ng/ml) and LY294002 (5 µM) or PD98059 (10 µM) for 6 h, and their migration rate was measured. The results showed that the addition of MAPK inhibitor completely blocked FABP4-induced HCASMC migration at 6 h (p<0.05, Figure 2D). Together with the partial inhibition of migration observed with the PI3K inhibitor (LY294002), these results indicate that the MAPK pathway is the main pathway responsible for the FABP4-mediated increase in cell migration. 

No cytotoxic effect, measured as LDH leakage, was observed in any of the experiments performed. 

### Effect of FABP4 on ERK1/2 and AKT activation in human coronary artery smooth muscle cells (HCASMCs)

**Figure 3:**
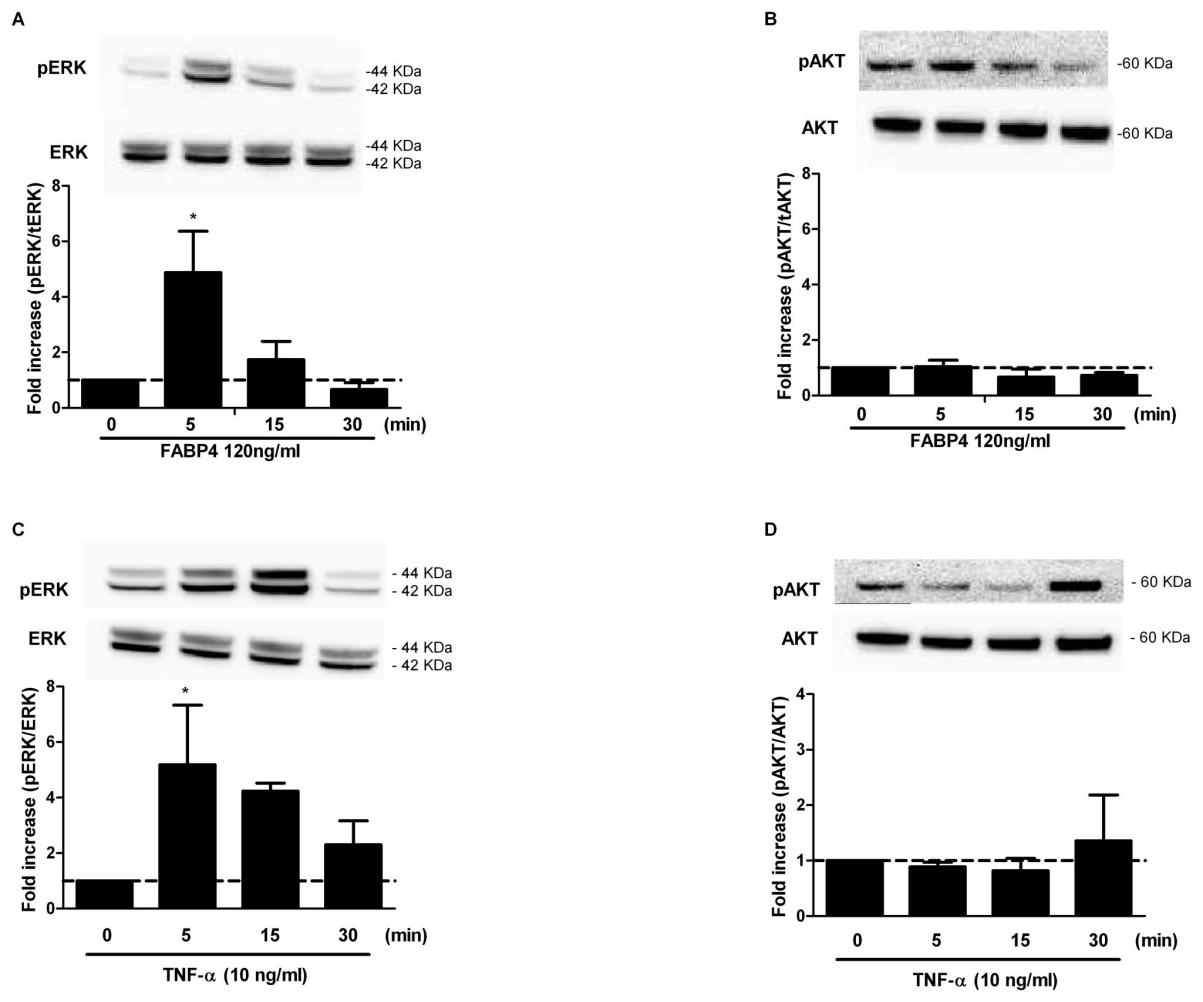
Effect of FABP4 (120 ng/ml) on ERK1/2 and AKT activation in HCASMCs. Effect of FABP4 (120 ng/ml) on ERK1/2 (A) and AKT (B) activation in HCASMCs. TNF-α (10 ng/ml) was used as the positive control (C, D). Representative western blots and relative densitometric analyses are shown. The results are expressed as the mean ± SEM of three separate experiments. *p<0.05 vs. unstimulated cells (control).

As reported in previously published literature, VSMC proliferation and migration are mediated via the ERK and Akt pathways[2,3]. Therefore, the effects of FABP4 on the activation of ERK1/2 and Akt were explored. Cells were incubated with FABP4 (120 ng/ml) for 5, 15 or 30 min, and the total cell extracts were analysed. The results indicated that FABP4 rapidly and significantly activated ERK1/2 phosphorylation in as little as 5 min (4.8-fold increase, p<0.05); the phosphorylation returned to basal levels after 30 min (Figure 3A). As shown in Figure 3B, FABP4 had no effect on AKT activation. TNF-α (10 ng/ml), which was used as a control, activated ERK1/2 and AKT phosphorylation at 5 and 30 min, respectively (5.2 and 1.35-fold increases, respectively) (Figure 3C).

We next analysed whether extracellular FABP4 affects the cellular pool of FABP4. Figure S1 shows that the incubation of HCASMCs with 120 ng/ml FABP4 for 5, 15 or 30 min increased the cellular pool of FABP4 2-fold compared to non-treated cells (p<0.05).

### Effect of FABP4 on transcription factors activated by the MAPK signal transduction pathway in human coronary artery smooth muscle cells (HCASMCs)

**Figure 4:**
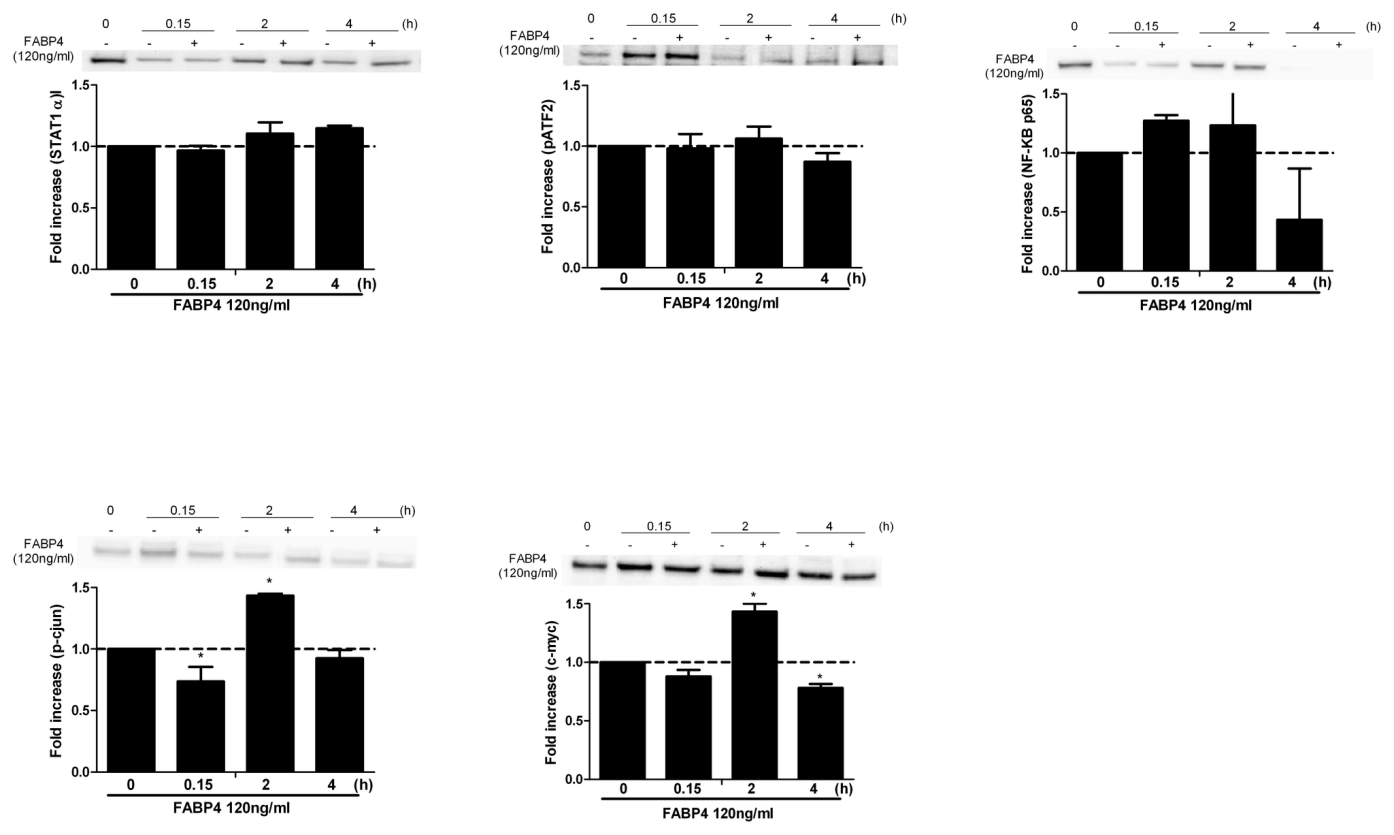
Effect of FABP4 (120 ng/ml) on STAT1α, ATF2, NF-Kb p65, c-jun and c-myc activation in HCASMCs. Representative western blots and relative densitometric analyses are shown. The results are expressed as the mean ± SEM of three separate experiments. *p<0.05 vs. unstimulated cells (control).

**Figure 5:**
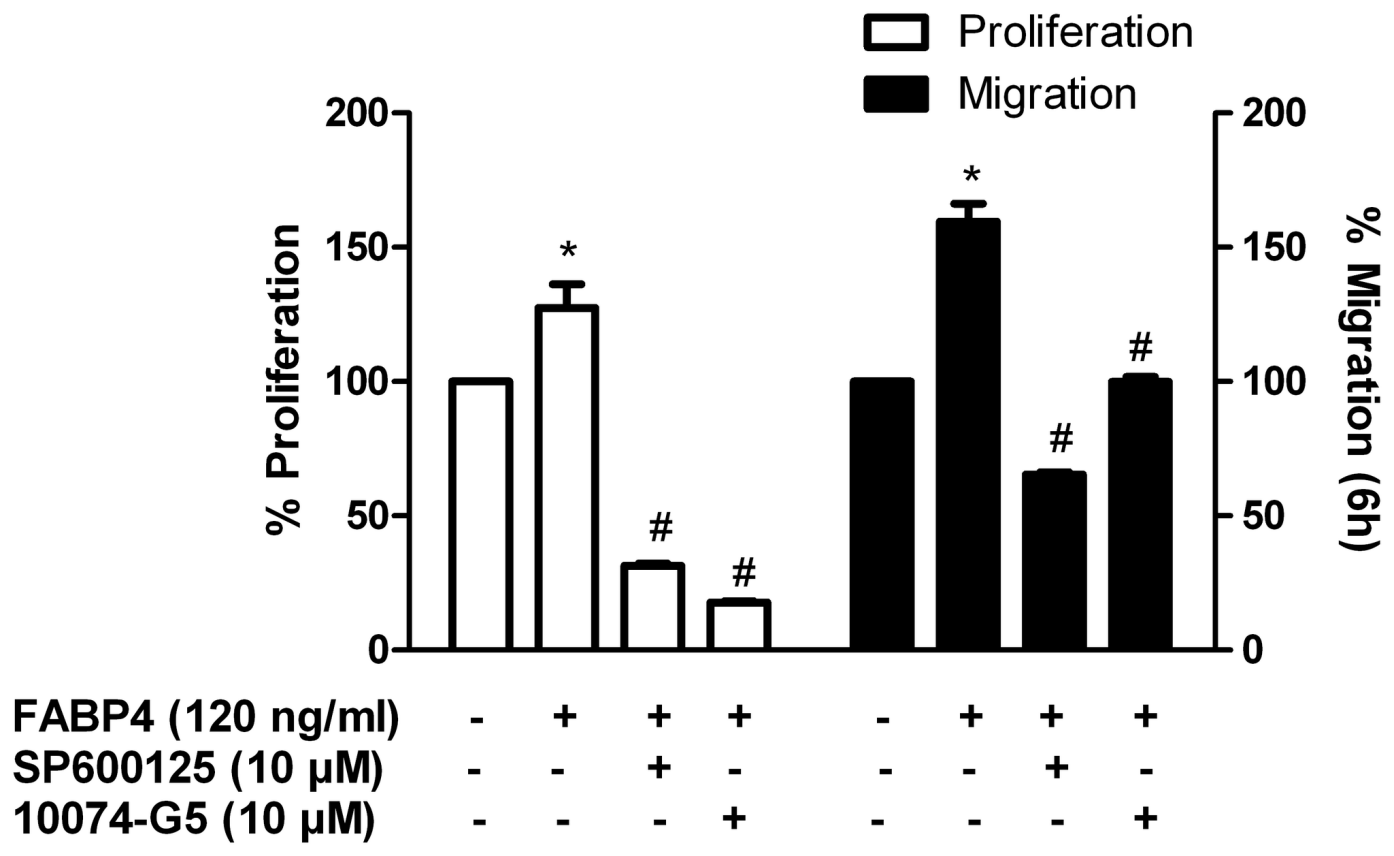
Effects of the c-jun inhibitor (SP600125) and the c-myc inhibitor (10074-G5) on FABP4-induced HCASMC proliferation and migration. Effect of SP600125 (10 µM) and 10074-G5 (10 µM) on FABP4 (120 ng/ml)-induced HCASMC proliferation at 24 h and on cell migration at 6 h. The results are expressed as mean±SEM of three separate experiments run at least in triplicate. *p<0.05 vs unstimulated cells; #p<0.05 vs FABP4-stimulated cells.

Next, we investigated the effect of FABP4 on the transcription factors activated by the MAPK signal transduction pathway. HCASMCs were incubated with FABP4 (120 ng/ml) for 15 min, 2 h or 4 h, and the nuclear cell extracts were isolated. We analysed the effect of FABP4 on the active forms of the following transcription factors: phospho-ATF2 (Thr^71^), phospho-c-jun (Ser^73^), c-myc, STAT1α and NF-kB p65. As shown in Figure 4, the nuclear extracts of the cells incubated with FABP4 (120 ng/ml) for 2 h tended to yield increases in the active forms of all of the transcription factors analysed, with increases in the active forms of c-jun (1.43±0.01, p<0.05) and c-myc (1.43±0.06, p<0.05) reaching statistical significance. To confirm the involvement of the transcription factors c-jun and c-myc in FABP4-induced HCASMC proliferation and migration, HCASMCs were incubated with FABP4 (120 ng/ml) and SP600125 (10 µM) or 10074-G5 (10 µM) for 24 h and BrdU incorporation was measured. We found that SP600125 and 10074-G5 significantly attenuated FABP4-induced HCASMC proliferation (p<0.05) (Figure 5, left axis). In another experiment, HCASMCs were incubated with FABP4 (120 ng/ml) and SP600125 (10 µM) or 10074-G5 (10 µM) for 6 h, and their migration rate was measured. Both inhibitors blocked the stimulatory effect of FABP4 on cell migration (p<0.05) (Figure 5, right axis). We conclude that the activation of both c-jun and c-myc is involved in FABP4-induced cell proliferation and migration.

### Effect of FABP4 on the expression of cyclin D1, MMP2, CCL2, and fibulin 4 and 5

**Figure 6:**
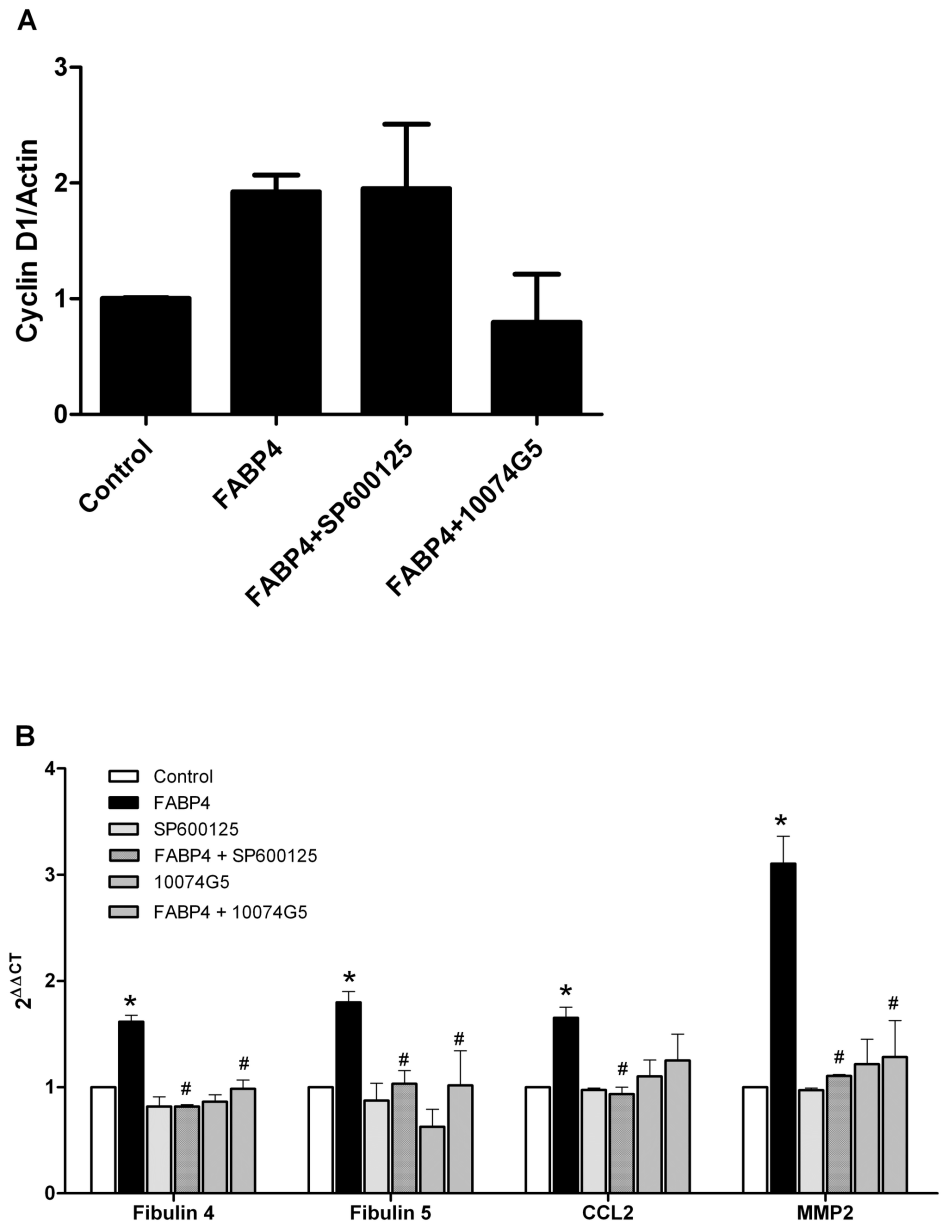
Effect of FABP4 on target genes regulated by c-fos and/or c-myc. (A) Effect of FABP4 on cyclin D1 expression. Proliferating HCASMC were incubated with 120 ng/ml FABP4 in the presence or absence of c-myc (10074G5) and c-jun (SP600125) inhibitors for 6 h. Western blotting was performed to evaluate the expression of cyclin D1 in the cells, with actin as an internal control. (B) Migrating HCASMC were incubated with 120 ng/ml FABP4 in the presence or absence of c-myc (10074G5) and c-jun (SP600125) inhibitors for 24 h. Total RNA was extracted from the cells, and the expression of fibulin 4, fibulin 5, MMP2, and CCL2 was measured by quantitative RT-PCR using TaqMan primers and the 2ΔΔCt method. *p<0.05 vs unstimulated cells; #p<0.05 vs FABP4-stimulated cells.

Cyclin D1 and MMP2, CCL2, and fibulin 4 and 5, which are involved in cell cycle regulation and cell migration, respectively, are downstream genes of the c-Fos and c-myc transcription factors. To investigate the effect of FABP4 on cyclin D1 expression, proliferating HCASMC were incubated with 120 ng/ml FABP4 in the presence or absence of the inhibitors 10074G5 (10 µM) or SP600125 (10 µM) for 6 h. The results showed that FABP4 increases cyclin D1 expression 1.9-fold (Figure 6A). The addition of the c-jun inhibitor SP600125 had no effect on FABP4-induced cyclin D1 expression, while the addition of the c-myc inhibitor 10074G5 caused the expression of cyclin D1 to return to basal levels (Figure 6A). 

To investigate the effect of FABP4 on fibulin 4, fibulin 5, MMP2, and CCL2 expression, migrating HCASMC were incubated with 120 ng/ml FABP4 in the presence or absence of 10074G5 (10 µM) and SP600125 (10 µM) for 24 h. The results showed that FABP4 significantly increased the mRNA levels of all genes studied (p<0.05); the greatest effect was observed for MMP2 mRNA, the level of which increased 3-fold (3.1±0.2, p<0.05)(Figure 6B). The addition of 10074G5 (10 µM) and SP600125 (10 µM) reversed this effect for all the genes studied (p<0.05) (Figure 6B).

## Discussion

Increases in VSMC proliferation and migration are key events in the pathogenesis of atherosclerosis, as well as intimal hyperplasia after vascular injury[32]. FABP4 exhibits biological activity in various cell types to promote a proinflammatory state and vascular dysfunction[7]; however, nothing was previously known about the direct influence of FABP4 on HCASMC activation and the intracellular signalling pathways involved. In the present study, we demonstrated that FABP4 directly promotes the in vitro proliferation and migration of HCASMCs through the activation of the ERK1/2 MAPK signalling pathway. We also demonstrated that FABP4 activates the nuclear transcription factors c-myc and c-jun and that it increases the expression of their downstream genes cyclin D1 and MMP2, CCL2, and fibulin 4 and 5, which are involved in cell cycle regulation and cell migration, respectively. 

It is well known that FABP4 is an adipocyte- and macrophage-produced protein that promotes insulin resistance, hypertriacylglycerolaemia and atherosclerosis and is also a circulating protein, the levels of which are independently and positively associated with metabolic syndrome and vascular disease. Data from animal studies also support the pathogenic role of FABP4 in cardiovascular disease[33,34]. However, little is known concerning the role of FABP4 as a secreted adipokine, including the target tissues, the actions of the protein and the underlying mechanisms. FABP4 might be released into the circulation at least in part by adipocyte-derived microvesicles or from the lysis of large adipocytes[8]. Our results support a direct effect of extracellular FABP4 on peripheral tissues, specifically on HCASMCs. There are few studies indicating a direct effect of FABP4 on cells. Lamounnier-Zepter et al. demonstrated that recombinant FABP4 reduces the contractile capacity of cardiomyocytes, illustrating a cardiodepressant activity of the factor[26]. In addition, we have previously shown that exogenous FABP4 impairs endothelial function by inhibiting the activation of the insulin signalling pathway, resulting in decreased eNOS activation and NO production[29]. 

Our present results demonstrate that recombinant FABP4 induces dose-dependent HCASMC proliferation. We found that this effect is specific because the addition of an anti-FABP4 antibody inhibited the cellular proliferation. In addition to cell proliferation, we found that FABP4 also increases cell migration in a dose- and time-dependent manner. Our findings are in good agreement with a recently published study showing that recombinant and secretory FABP4 cause an enhancement of growth and migration in human aortic smooth muscle cells through a ROS-mediated mechanisms[35]. Using another cell type, Elmasri et al. revealed a novel pro-angiogenic role for endothelial cell FABP4, which promoted the migration and invasion of HUVECs[36]. The authors showed that FABP4 deficiency alone was sufficient to induce apoptosis in HUVECs. Furthermore, the knockdown of FABP4 dramatically reduces the proliferation of endothelial cells both under baseline conditions and in response to VEGF[24]. The induction of cultured HCASMC migration and proliferation by FABP4, as demonstrated in the current study, suggests a potential role for this adipokine in promoting vascular pathology. 

We studied different mechanisms for FAPB4-induced HCASMC proliferation and migration. The involvement of the ERK1/2 MAPK cascade and the PI3K/AKT signalling pathway in SMC proliferation and migration has previously been demonstrated[2,3]. The c-Raf/MEK/ERK pathway is essential for cell proliferation and migration[37], and our results indicate that FABP4 increases the phosphorylation of ERK1/2, which suggests that FABP4 utilises the ERK pathway to induce smooth vascular cell proliferation and migration. We found that although FABP4 had no effect on Akt activation, an inhibitor of PI3K (LY294002) blocked the effects of FABP4 on HCASMC proliferation and migration, which suggests the activation of an additional kinase pathway. Crosstalk between the Ras/c-Raf/MEK/ERK pathway and other signalling pathways appears to occur. For example, RAS can activate the PI3K/AKT pathway, in addition to having other shared inputs, and there appears to be some compensation for the loss of signalling activity when one pathway or the other is inhibited. Moreover, the selective inhibition of MAPK kinase by PD98059 significantly reduced FABP4-induced HCASMC proliferation and migration. Taken together, these data clearly demonstrate that FABP4-induced HCASMC proliferation and migration are dependent on ERK1/2 and PI3K activation without the direct involvement of Akt activation. The translocation of phosphorylated ERK to the nucleus can activate transcription factors such as c-myc and c-jun, and this type of activation was demonstrated in the present study. Thus, our results suggest that the signals initiated by FABP4 activate the c-Raf/MEK/ERK pathway, resulting in the induction of the c-myc and c-jun transcription factors. Although the observed increases in the levels of these transcription factors were not striking, the addition of specific inhibitors of c-jun (SP600123) and c-myc (10074G5) significantly attenuated FABP4-induced HCASMC proliferation and migration. It has been widely observed that modest changes on transcription factor levels can have a great impact on the concentrations regulated proteins. Cyclin D1 and MMP2, CCL2, and fibulin 4 and 5 are downstream genes of c-Fos and c-Myc transcription factors and are involved in cell cycle regulation and cell migration, respectively. In this study, we found that the treatment of HCASMCs with FABP4 increased cyclin D1 expression and cell proliferation through c-myc, as demonstrated by the addition of inhibitors of c-jun and c-myc. This effect was more striking when we evaluated the expression of genes involved in cell migration (MMP2, CCL2, fibulin 4 and 5). Thus, it is conceivable that increases in MAPK signalling by extracellular FABP4 lead to increased levels of cell migration-related proteins rather than increased levels of proteins related to cell proliferation. 

It is not known whether extracellular FABP4 is internalised into the cell or whether it acts by an intracellular mechanism. We observed that the cellular pool of FABP4 increased after FABP4 incubation, which suggests that FABP4 can bind to the cell membrane of HCASMCs and/or be internalised into the cells. 

The concentrations of FABP4 used in the present study are higher than the levels observed in vivo. The FABP4 doses used in our study were those that we found to have a biological effect on the insulin-dependent nitric oxide pathway in HUVEC[29]. Notably, the concentrations of recombinant FABP4 used in other in vitro studies were ~15 to 100 times higher than the concentrations used in our study. Lamounier-Zepter et al.[26] used ~1500 ng/ml of FABP4 to show an effect in cardiomyocytes, and Lu et al.[35] used 2500 to 10000 ng/ml of FABP4 to show an effect in aortic SMCs. Thus, it seems that higher concentrations of FABP4 than those observed in vivo are required to produce significant effects in vitro. In addition, we cannot rule out that the concentration of a biomarker, in this case FABP4, found in the interstitial tissue was higher than the concentration in plasma.

In conclusion, our results demonstrate that exogenous FABP4 induces VSMC migration and proliferation *in vitro*, mainly via ERK activation. Our findings suggest that high levels of FABP4 in the circulation are not simply a clinical manifestation of cardiometabolic risk but are also a causative factor in vascular pathology. Furthermore, the data from our group and other groups suggest that pharmacological inhibition of FABP4 should be explored as a potential therapeutic strategy for treating atherosclerosis and reducing cardiovascular risk. 

## Supporting Information

Figure S1 **Presence of FABP4 in total cell lysates.** HCASMCs were treated with or without FABP4 (120 ng/ml) for the indicated times. Representative blots are shown. The data represent the mean ± SEM values obtained in three independent experiments. *P<0.05 vs. without FABP4.
(TIF)
