# Correction: FABP4 Induces Vascular Smooth Muscle Cell Proliferation and Migration through a MAPK-Dependent Pathway

**DOI:** 10.1371/journal.pone.0146632

**Published:** 2016-01-05

**Authors:** Josefa Girona, Roser Rosales, Núria Plana, Paula Saavedra, Lluís Masana, Joan-Carles Vallvé

The following information is missing from the Funding section: This work was supported by grants from Fondo Europeo de Dessarrollo Regional (FEDER).
