# Supplementary figures and images for: FABP4 Induces Vascular Smooth Muscle Cell Proliferation and Migration through a MAPK-Dependent Pathway
Source: PLoS One. 2013 Nov 29;8(11):e81914. doi: 10.1371/journal.pone.0081914 (PMC3843707; doi:10.1371/journal.pone.0081914)

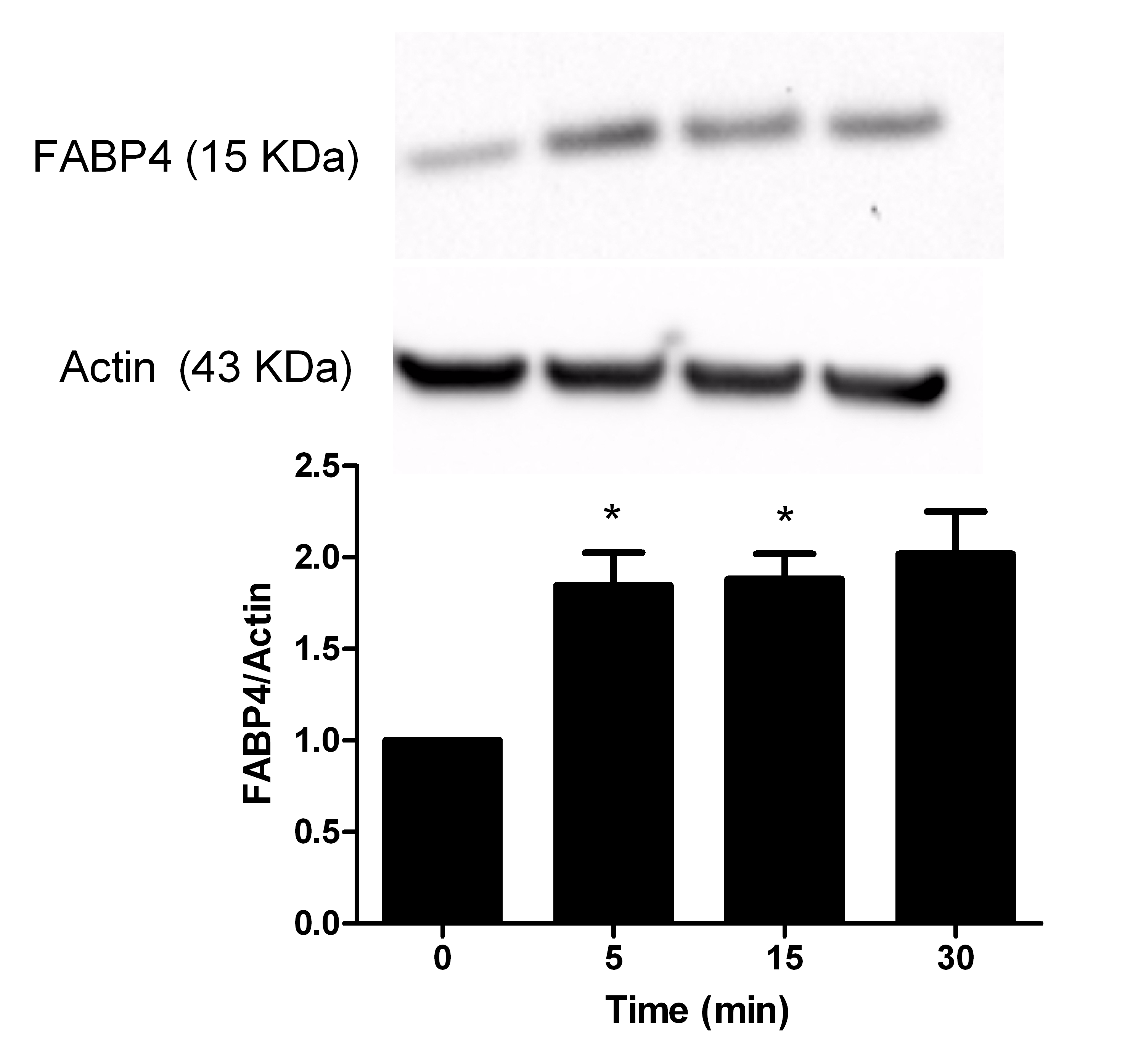

Supplement: Figure S1 — Presence of FABP4 in total cell lysates. HCASMCs were treated with or without FABP4 (120 ng/ml) for the indicated times. Representative blots are shown. The data represent the mean ± SEM values obtained in three independent experiments. *P<0.05 vs. without FABP4. (TIF) [file pone.0081914.s001.tif]
